# Identifying Local and Centralized Mental Health Services—The Development of a New Categorizing Variable

**DOI:** 10.3390/ijerph15061131

**Published:** 2018-05-31

**Authors:** Taina Ala-Nikkola, Sami Pirkola, Minna Kaila, Grigori Joffe, Raija Kontio, Olli Oranta, Minna Sadeniemi, Kristian Wahlbeck, Samuli I. Saarni

**Affiliations:** 1 Clinic of Psychiatry and Clinic of Public Health Välskärinkatu 12 and Stenbäckinkatu 9, University of Helsinki and Helsinki University Hospital, FI-00029 Helsinki, Finland; minna.kaila@helsinki.fi (M.K.); grigori.joffe@hus.fi (G.J.); raija.kontio@hus.fi (R.K.); minna.sadeniemi@helsinki.fi (M.S.); 2 Unit for Mental Health, National Institute for Health and Welfare (T.H.L.), Mannerheimintie 168, FI-00270 Helsinki, Finland; kristian.wahlbeck@thl.fi; 3 Public Health Medicine, University of Helsinki and Helsinki University Hospital, FI-000014 Helsinki, Finland; 4 University of Tampere School of Health Sciences and Tampere University Hospital, Lääkärinkatu 1, FI-33014 Tampere, Finland; sami.pirkola@staff.uta.fi; 5 University of Turku, FI-20014 Turku, Finland; 6 Lohja Hospital Area, Sairaalakatu 8, 08200 Lohja, Finland; 7 Turku University Hospital and University of Turku, Kiinanmyllynkatu 4-8, FI-20520 Turku, Finland; olli.oranta@tyks.fi (O.O.); Samuli.saarni@gmail.com (S.I.S.); 8 Department of Social Services and Health Care, City of Helsinki, FI-00099 Helsinki, Finland

**Keywords:** mental health care, health service research, integrated care, European Service Mapping Schedule-Revised

## Abstract

The challenges of mental health and substance abuse services (MHS) require shifting of the balance of resources from institutional care to community care. In order to track progress, an instrument that can describe these attributes of MHS is needed. We created a coding variable in the European Service Mapping Schedule-Revised (ESMS-R) mapping tool using a modified Delphi panel that classified MHS into centralized, local services with gatekeeping and local services without gatekeeping. For feasibility and validity, we tested the variable on a dataset comprising MHS in Southern Finland, covering a population of 2.3 million people. There were differences in the characteristics of services between our study regions. In our data, 41% were classified as centralized, 37% as local without gatekeeping and 22% as local services with gatekeeping. The proportion of resources allocated to local services varied from 20% to 43%. Reclassifying ESMS-R is an easy way to compare the important local vs. centralized balance of MHS systems globally, where such data exists. Further international studies comparing systems and validating this approach are needed.

## 1. Introduction

The global ongoing reforms in mental health and substance abuse services (MHS) are defined in terms of balancing and integrating institutional and community care. The balance of services is being moved from hospitals, so that most services are provided in community settings close to the populations served, and hospital stays are reduced as far as possible. The integration of health services means that mental health services should be functionally integrated with other services; for example, mental health with primary care, and acute wards within general hospitals [1].

The evidence suggests that compared to hospital-centered systems, community-based health systems reach more patients [2,3,4,5], human rights are better respected [6,7,8], and de-stigmatization is achieved [9,10]. Diversified community-based MHS structures are associated with lower suicide rates than traditional hospital-based systems [11]. Community-based services are generally, although not always, more easily accessible to patients, and without gatekeeping, and the peer-support that is often offered by third-sector providers is more respecting of patient’s autonomy and self-determination, thus furthering de-stigmatization [7,12,13]. In addition, a more preventive approach is often seen as more cost-effective in the long run than traditional institutional and inpatient-based systems [5]. Community-based care has been found to be cost effective if the quality of institutional care is simultaneously developed [1,14,15]. In addition to mental health services, patients also need physical health services and social support (for education, work, accommodation). Meeting this need requires an integrated system in which community level primary services, secondary level specialized services, and tertiary level services function as a whole [5,16,17]. The fragmentation of physical and mental health services needs to be reversed for improved equality and outcomes of care for persons with mental disorders. Barriers such as administrative, financial and clinical hurdles need to be identified before successful integration of MHS. Kilbourne et al. (2008) suggested that strategies to overcome barriers to integrated care may require cooperation across different organizational levels, including administrators, providers and health care financers in order for integrated care to be established and sustained over time [18].

An adequate division of responsibilities between secondary and primary care (vertical collaboration and integration) and between social and health care organizations (horizontal integration) are also needed in Finland, where major reforms of social and health services are planned. (http://alueuudistus.fi/en/frontpage) [19,20]. On a horizontal scale, organizations operate within their own substance area (health service, social service or mental health service) at the same level of specialization, and value expansion is achieved by cooperation. On the vertical scale, the level of specialization increases when moving to the next stage of organization, e.g., from primary care to the secondary or tertiary care level. The reform aims to fully integrate all social and health services into user-oriented services, in effect (at least in theory) making the traditional organizational divisions between primary care, secondary care and social care obsolete. In general, such development could provide a more comprehensive and more easily manageable service structure, regardless of the funding and steering option. The MHS cost should be seen as a whole, because intensive local services could be more expensive than even long-term hospital care, but may still be seen as more cost-effective because of better outcomes. The local administrators and budget controllers need to engage in joint planning in order to develop effective and cost-effective care [21]. Investigating mental health system structures and their relationship to health outcomes is important. As this is often also very complex, investigating the effects of organizational sub-components (such as integration of care) on health, or the effects of strategy-level decisions on organizational sub-components or other intermediate outcomes (such as resource shifts), is important. Some service settings can even be viewed as treatments (e.g., “partial hospitalization”), whereas some treatments are always embedded in a service matrix (e.g., assertive community treatment) or organizationally combined (e.g., “integrated treatment” for co-occurring mental disorder and substance abuse). Ideally, for example, studies would focus on horizontal and vertical integration, primary care vs. secondary care, and local vs. centralized mental health authorities—Each of which could be conceptualized as a health care technology, with empirical studies to assess its effectiveness. [22]. All these studies need comparable, reliable instruments for classifying health care systems.

More generally, for all evidence-informed reforms, a fact-based view of the current state of affairs must be obtained, along with a view of the future and measurement instruments that can indicate progress. Thus, an instrument is needed that can be used to track changes in mental health services, including the balance between community (local) and centralized services. The European Service Mapping Schedule-Revised (ESMS-R) was designed to map mental health services, to describe their major characteristics, the provision of services, as well as resource allocation. The ESMS-R instrument allows for a good-quality common description of the socioeconomic profile of the population of a specified area, alongside key features of mental health service provision, including those provided by primary care and social services [17,23,24,25]. The first version of the ESMS has been used previously in Finland [11,26] and other European countries [27,28,29] as well as in Chile [30].

However, the ESMS-R as a mapping tool does not differentiate between services that should be provided locally and those that can be centralized, or between services without gatekeeping and those where gatekeeping is used. This kind of information would be valuable when reforming the organizations that provide health and social care, as is currently planned in Finland. We thus set out to develop a definition to differentiate local services and to apply that categorization to the ESMS-R instrument. In the study area, centralized services are mainly organized by hospital districts or specialized private or third sector organizations. Local services can be reached with or without gatekeeping, referral or other prior specialist consulting. All services can be organized by public, private or third sector (e.g., foundations or associations) organizations. 

The specific aims of the study were:

1. To create a new coding variable for the ESMS-R mapping tree to categorize MHS into local and centralized categories, for the use of developmental activities in different settings globally.
2. To test the feasibility of this new variable as a potential quality indicator for MHS, using a Finnish dataset representing a publicly managed Western service.
3. We also set out to test whether a quality indicator for MHS could be developed for ESMS-R, based on the hypothesis that when more MHS without gatekeeping are locally available, less centralized services are required.

## 2. Methods

The study methods consist of two parts: creation of the new coding variable for ESMS-R and then testing the new “Local service” variable. We used a modified Delphi procedure with alternating theoretical meetings and practical classification phases [31,32,33] to create the new variable, and a Finnish dataset covering a population of 2.3 million within 13 different catchment areas for testing. The creation process is reported in Methods and feasibility testing in Results.

### 2.1. The European Service Mapping Schedule-Revised Instrument and Dataset

The ESMS-R is derived from the previous European Service Mapping Schedule and the Description and Evaluation of Services and Directories for Long-Term Care in Europe coding system [17,23,34]. In the used version of ESMS-R, mental health services are classified into 89 different “main types of care” (MTC). The MTC is the main descriptor of the care function (for example, mobile acute team or acute hospital care). The MTCs are organized according to the “basic stable input of care” (BSIC), such as the organizational units that provide the services (for example, an acute ward or a day care center). The MTCs are categorized into six main branches: information for care (I), accessibility to care (A), self-help and voluntary help (S), outpatient care (O), day care (D), and residential care (R) [23,35]. The whole ESMS-R system is described in Supplementary Figure S1. 

We used the ESMS-R data collected by trained researchers from Southern Finland between 2012 and 2014. The data collection has been described previously [24,25,36]. Briefly, the study area included four hospital districts: Helsinki and Uusimaa, Carea, Etelä-Karjala, and Varsinais-Suomi. The districts are further divided into thirteen non-overlapping health care areas. The size of the adult population varied within the areas, from 18,200 to 500,000 inhabitants, the median being 128,000. In total, the study area covers 2.3 million people, with 1.8 million adults (18+), in 67 municipalities and representing 43% of the Finnish population. The study area is much more densely populated than the average for the whole country (174 vs. 16 inhabitants per square kilometer). Each study area has its own psychiatric in-hospital care, with some coordination at the hospital district level. Psychiatric hospital care is integrated into general hospitals in some study areas, but most areas still have free-standing psychiatric hospitals. 

The ESMS-R service mapping covers all municipalities in the study area and is aimed to include all adult (18+) mental health and substance use services (in primary, secondary and tertiary health care) and social services located in the catchment areas. The personnel resources allocated to each service unit were measured based on full-time equivalents (FTE). The services were classified by their vertical organizational level: primary care, secondary care or integrated care. Horizontally, the services were classified by their legal status: public, third sector, or private companies [24,35]. 

### 2.2. Creating the New “Local Service” Variable on Local Versus Centralized Services (Part One)

A modified Delphi technique [31,33] was used to develop the criteria for local services and the new “local service” variable for the ESMS-R service mapping system. The Delhi technique is used in various fields and is suitable e.g., for policy determination, such as decisions concerning which services are better arranged locally, with or without gatekeeping. The Delhi process in this study concentrated on seeking consensus for ESMS-R service classification in live meetings and independent practice classifications.

We invited an expert panel consisting of eleven mental health professionals (researchers and administrators) familiar with the ESMS-R, the ongoing research project and the current Finnish MHS system. The panel consisted of two senior administrative psychiatrists, two senior administrative nurses, a research professor, and researchers with work experience in the areas under study (Supplementary Table S1).

In the first part of the study the panel worked in four phases:

Phase 1. Two theoretical meetings
Phase 2. Individual classification (round one)
Phase 3. Consensus meeting and practical classification
Phase 4. Individual classification (round two) and final decisions

#### 2.2.1. Phase One: Theoretical Meetings

Two meetings were held to discuss how “local” and “centralized” services should be conceptualized and defined in MHS. The main idea was that services that are needed often should be arranged in local settings, in contrast with services that are needed seldom or need more resources or special equipment, which are better to arrange in a centralized setting. The background material included a draft developed by the Ministry of Social Affairs and Health for a new legal conceptualization of local and centralized health and social services [19]. 

The baseline proposal for the panel was made by T.A.N., K.W. and S.S., suggesting that all services on the main branches of “information for care”, and “accessibility to care” should be classified as local services, and all services on the main branch “residential care” should be classified as centralized services. Thus, only services under the branches “self-help and voluntary care”, “outpatient care” and “day care” would need to be re-classified. 

**Figure 1:**
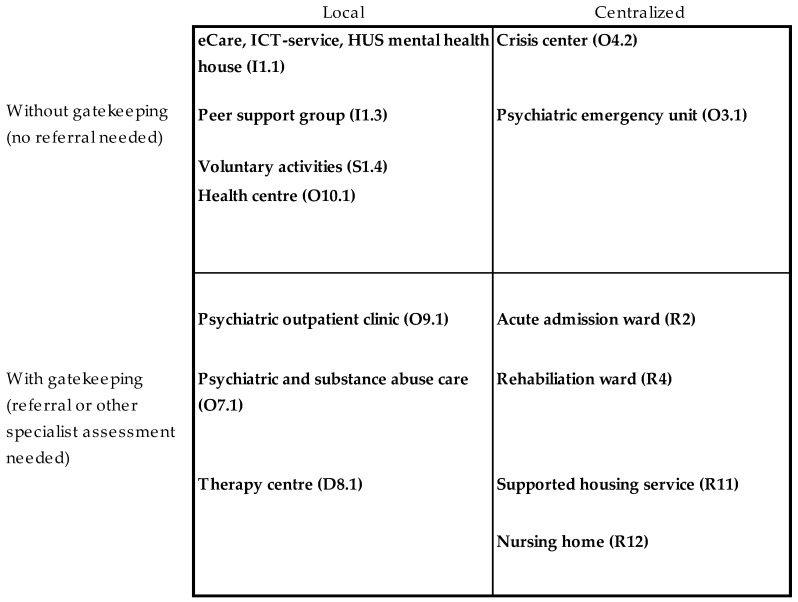
Quadrangle for ESMS-R* main types of care (MTC) new variable classification (Round One). *European Service Mapping Schedule–Revised.

The first theoretical meeting concluded that the initial definition of local versus centralized MHS was insufficient, as it did not recognize the potential difference between organizations or patients’ viewpoints regarding local or centralized services. The second theoretical meeting elaborated on this and concluded that in addition to physical locality, other factors influencing access to services should be considered. Thus, two new viewpoints should be addressed: (1) the needs of patients to access services with no or low barriers to access (distance, gatekeeping, costs etc.) and (2) the needs of organizations to recognize some services as so complex, rare or expensive that they require a specialist assessment or some other kind of gatekeeping. Thus, two categories were added: “local services with gatekeeping” and “centralized services without gatekeeping”, changing the initial dichotomy into four categories (Figure 1). This classification was accepted for empirical testing. It was agreed that “residential care” would be classified as centralized services with gatekeeping. 

#### 2.2.2. Phase Two: Individual Classification (Round One)

The quadrangle shown in Figure 1 was used as the basis for re-classifying the 46 (*n* = 89) types of services in ESMS-R branches O and D and validating the baseline proposal on ESMS-R branches I, A and S-H. The baseline proposal on ESMS-R branch R (centralized with gatekeeping) was agreed without re-classification. Six panelists participated in the exercise. The panelists were in 100% agreement on the category “information for care”, in 90% agreement on “self-help services” and in 80% agreement on “accessibility to care”. Their responses were more variable regarding the “outpatient” (*n* = 24) and “day care” main branches (*n* = 22) (Supplementary Table S2).

#### 2.2.3. Phase Three: Consensus Meeting and Preliminary Testing of the Classification 

The aim of the Delphi meeting was to reach consensus and make final decisions on how the 89 MTCs should be divided into the four categories. The classifications in which at least half of the experts agreed were accepted and only the remaining 14 of 89 MTC were further discussed (Supplementary Table S3). Discussions were continued until consensus was reached.

**Figure 2:**
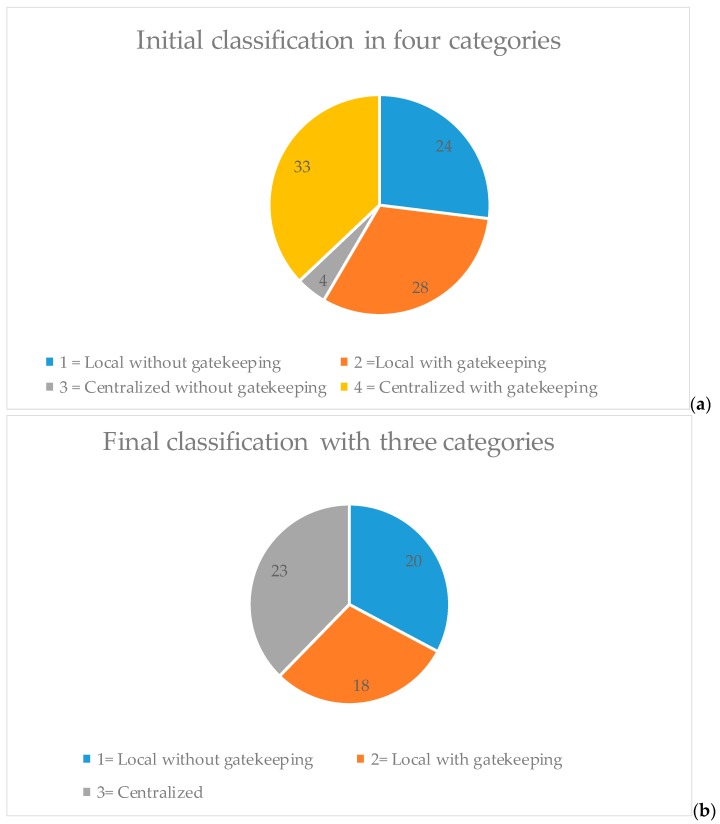
Proportions of different main types of care (MTCs) classified as local or centralized; initial and final classifications. Initial classifications (**a**) and final classifications (**b**) represented

The resulting classification divided the different MTCs relatively equally between centralized with gatekeeping (37%), local services with gatekeeping (31%), and local services without gatekeeping (27%). However, only four MTC were classified in the category centralized services without gatekeeping (5%) (Figure 2a). Investigation of the empirical dataset consisting of 987 organizational units (i.e., BSICs) indicated that centralized services with gatekeeping were most frequent (40.1%), followed by local services without gatekeeping (37%), local services with gatekeeping (19%) and centralized services without gatekeeping (3%) (Supplementary Table S3).

#### 2.2.4 Phase Four: Individual Classification (Round Two) and Final Decisions

The results of the classification were considered problematic, as there were only four types of care (ESMS-R codes 0.3.1, 0.3.2, 0.4.1 and 0.4.2) that were classified as centralized services without gatekeeping. Furthermore, it was considered that centralization of services can often in itself effectively result in gatekeeping, caused for example by the distance to services. The panel decided that these services should be merged into the other three categories. A second individual classification round was organized to reclassify these four types of care (Shown with red colour in Supplementary Table S2).

The final local or centralized services variable thus included three categories: (1) local services without gatekeeping, (2) local services with gatekeeping and (3) centralized services (Figure 2b). The final classification of every ESMS-R MTC is shown in Supplementary Figure 1. In sum, MTCs belonging to the “information for care”, “accessibility to care” and “self-help and voluntary care” categories were mostly classified to local services without gatekeeping (7/9, 3/5 and 7/10, respectively), “outpatient care” to local services with gatekeeping (19/24) and “day care” and “residential care” to centralized services (12/22 and 19/19, respectively).

### 2.3. Testing the New European Service Mapping Schedule-Revised-Local Service Variable (Part Two)

The practical usability of the new ESMS-R-Local service variable was tested by addressing the following questions in the dataset:

1. The balance between local and centralized services was explored by comparing:
  (a) The proportion of service units classified as local or centralized.
  (b) The proportion of resources measured as full-time equivalents allocated to local services.
2. The differences in proportion regarding services provided as local without gatekeeping (in BSIC and FTE) between the areas were explored and considered as a quality indicator.
3. The types of services provided by public (primary or secondary health care), private, or third sector providers were explored in order to estimate how different types of local or centralized services integrate horizontally and vertically with other health services.

Descriptive statistics were used to explore associations, while Spearman rank correlations and linear regression modeling were used for analyzing associations.

## 3. Results: Testing the New “Local Service” Variable

### 3.1. The Balance between Local and Centralized Services

Our dataset included services classified into 61 different MTCs (out of the 89 different possibilities in the used version of ESMS-R) delivered at 986 service units (BSIC) with a total full-time equivalent (FTE) personnel of 6785. The distribution of these BSICs and FTE to local or centralized services is shown in distribution of service units (BSICs) and mental health personnel (FTE) to local or/and centralized services Table 1. Of the service units identified, 41% were classified as centralized, 37% as local services without gatekeeping and 22% as local services with gatekeeping. Of the personnel, 67% worked in centralized services, 11% in local services without gatekeeping and 22% in local services with gatekeeping.

### 3.2. The Difference between Study Areas in the Proportion of Services Provided as Local Services without Gatekeeping as a Potential Quality Indicator

The full time equivalent (FTE) personnel per 1000 adults (18+) by provider status (a.), organizational level (b.), local versus centralized services (c.) is shown in Table 2 and the difference in service units (BSIC) is shown in Supplementary Table S4.

The personnel allocated to any local (with or without gatekeeping) services varied widely, from 0.31 per 1000 adults in the Carea study area to 1.08–1.13 persons per 1000 in Turku and Salo (Table 2). The proportion of total resources allocated to local services varied less, but the range was still high, from 20% to 43% (mean 31%). The number of personnel allocated to local services without gatekeeping again varied more, from 0.06 per 1000 (Kymenlaakso) to 1.01 (Salo) (mean 0.5). The proportion of total resources allocated to local services without gatekeeping varied from 1.4% (Kymenlaakso) to 30.5% (Salo) (mean 13.1%).

The most populous area (Helsinki) had the largest total number of BSICs (196), whereas Turunmaa had the smallest number (13). Conversely, the Salo and Carea areas had the highest numbers of service units relative to the population (0.8 units per 1000 adults), whereas the smallest number was found in the Jorvi area (0.3). There was variation between the areas in the number of local services without gatekeeping relative to the population (range 0.10 to 0.49, mean 0.25 per 1000 adults), local services without gatekeeping (0 to 0.26, mean 0.10) and centralized services (0.12 to 0.35, mean 0.23).

There were no significant associations between the number of personnel (per 1000 adults) and the proportion of personnel allocated to local services, or between the number of personnel and the number of local service units. 

Table 3 shows the correlations between allocated full time equivalents per 1000 18+ in local vs centralized services. There is a strong correlation between total personnel and personnel allocated to centralized services, but not with personnel allocated to other types of services. 

To investigate the issue further, linear regression models were created with total personnel resources as a dependent variable and the proportion of personnel allocated to centralized services as an independent variable, while controlling for population size, service needs indicator (mental health index) or both. The relative proportions of resources allocated to centralized services were not correlated with the total resources in the regression models, with or without controls. Only the mental health index correlated significantly with total resources. 

### 3.3. The Types of Services Provided by Public or Private Providers, Classified on the Basis of the Level of Horizontal and Vertical Integration with Other Health Services 

We explored how different types of local or centralized services were integrated horizontally and vertically by examining which types of services are provided by public, private or third sector providers and by the organizational level of specialization (primary or secondary care). The proportion of local vs, centralized services by provider status (a) and vertical level of organization (b) is shown in Table 4. The distributions by area were presented earlier in Table 2. 

The third sector produced mostly local services without gatekeeping (54% of third sector service units), although 30% of third sector service units were centralized services. The public providers produced all types of services, whereas private providers concentrated on centralized services, e.g., supported housing (87% of their units). By contrast, 66% of all local services without gatekeeping were produced by the third sector, whereas the public sector produced 60% of local services with gatekeeping. Regarding personnel, 65% worked in the public sector, and the remainder in the third (20%) and private sectors (15%). Concerning specialization, 78% of service units were categorized as specialized health care services. These services were divided almost equally into local services without gatekeeping (43%) and centralized services (39%). Primary care produced mostly centralized and local services with gatekeeping. Of the local services without gatekeeping, only 5% were provided by primary care (Table 4). On the basis of FTEs, the proportion of primary care was much greater, with just over 50% of the personnel.

## 4. Discussion

The aim of the study was to create a new coding variable for the ESMS-R mapping tree for classifying mental health services into local and centralized categories. The aim was to develop a potential quality indicator, based on the amount of services and resources available locally, for the use of integrative mental health reforms in different settings globally. Ideally, this could help to follow the development of mental health systems towards a balanced care model [1,37].

The modified Delphi-panel technique, including meetings with theoretical discussions, individual classification and consensus decision making, was used. Interestingly, after preliminary empirical testing, the baseline suggestion with a dichotomy of two categories of services (local and centralized) evolved via a quadrangle (local and centralized, with and without gatekeeping) into three categories: local services with or without gatekeeping, and centralized services. 

Results of testing the new variable, named ESMS-R-Local Service, in the dataset of 2.3 million adults living in 13 areas indicated a wide variation in absolute resources, but also relatively in resource allocations between local and centralized services. Personnel resources in centralized services appeared to correlate with total resource needs when using Spearman’s *ρ*. Total resources also correlated with objective needs (mental health index) for MHS, suggesting that the differences in centralized resources may be justified by need. This indicates that this kind of structured and classified recording of service structures would be useful in managing modern reform processes, regardless of their funding and steering settings. It has potential as a factor explaining certain positive outcomes regarding the quality of life of long term mental illness patients and the effects of early intervention.

Our study can be compared both to studies describing MHS classification systems, and those empirically exploring different systems. There are several general instruments for describing and assessing MHS systems and the need for mental health and disability services [38,39,40,41]. In addition to many disease- or patient group-specific instruments, The World Health Organization’s Assessment instrument for mental health systems (WHO-AIMS) has been developed on the basis of a large information and intelligence package, including input from many countries, international experts, and the WHO technical staff [42]. The tool consists of six domains: policy and legislative framework, MHS, mental health in primary care, human resources, public education and links with other sectors, and monitoring and research. The WHO-AIMS provides essential information within countries and helps develop fact-based mental health policy and service delivery, especially for low—and middle—income countries. [43]. The European service mapping schedule (ESMS) differs from WHO-AIMS in that the former is far more detailed and specific to MHS, making it a suitable instrument for integrating the individual-level outcomes and organizational-level structure description and evaluation data, especially in resource-rich settings [17,23]. In our study, residential services were all categorized to central services, although many individual accommodation services are arranged locally [44]. The systematic collection of information about coverage and the cost-effectiveness of MHS is important, especially when service structure reforms are planned and evaluated.

The ESMS has been used in many studies, from micro-area comparisons to cross-country evaluations [23,29,45]. In one comparative analysis in which Italy and Spain were compared, it was found that in Italy, innovative community service structure was associated with low hospital-bed use, high day-service use and contacts in the community services, whereas in Spain the hospital bed use was low despite the limited community-based services [27]. A previous version of the ESMS was used to compare northern Norway and Archangel County in Russia [29]. It found that more decentralized and differentiated services (with general practitioner (GP) gatekeeping supported by outpatient consultation) were associated with a decreased use of hospital care in Norway. In Russia, more resources were spent on institutional care, with few possibilities for outpatient care, especially in rural areas. The results were interpreted as supporting the importance of collaboration between mental health care and primary care, highlighting differences in resourcing between rural and urban areas [29]. Service differentiation, community orientation, and an emphasis on primary care appear to be significant when resources in centralized services are downsized. Our cross-sectional results also support this conclusion, suggesting that it may even be possible to decrease centralized resources and total resources simultaneously. However, both the above studies emphasized the fact that cultural aspects need to be considered when health care structures are compared. This is underlined by our comparisons [24,36] in a relatively small country showing local variation, most probably as the result of local health policy and the history of decision making rather than due to areal variation in mental health needs. 

Integration is considered to be the focus of service system research [22]. However, a recent systematic review on coverage of MHS found only seven studies, but suggested that coverage data could help optimize the limited resources and the balancing of local and centralized services, despite the scarcity of studies [46]. This reinforces our starting point of the need for more research into the associations between MHS properties and health outcomes. Reliable and valid instruments are needed to systematically evaluate and compare health systems within and between countries. 

### Strengths and Limitations

A strength of the study is the use of a standardized and internationally validated ESMS-R instrument for investigating service structure. Another strength is the high quality of the dataset with which to test the new ESMS-R-Local service variable. The data covered 43% of the adult population in Finland, including both larger cities and lower density areas and MHS organized by public, third sector and private providers on primary, secondary and tertiary levels. Moreover, comprehensive information on resources was available.

The study used a modified Delphi technique consisting of theoretical discussions, individual expert classifications and consensus decision making regarding the final classification. However, the individual classifications showed rather wide variation, most probably due to the complexity of the concept of gatekeeping. Therefore, the consensus discussions played a significant role, and the new classification variable should be considered as a prototype in need of further validation and testing. The expert panel could have been larger, more diverse and could also have included e.g., service users.

Regarding the empirical data, the transferability of health system results is always somewhat relative due to political, cultural and historical factors that influencing the systems. Even in Finland, the administrative structures of the 13 areas included in the study vary somewhat; some areas are independent hospital districts, some a portion thereof, and some are administered by a single large city. Obviously, the inclusion of only 13 areas limits the statistical power of the analyses.

## 5. Conclusions

The ESMS-R is a valuable and flexible instrument for evaluating variable MHS structures and their development needs, in contemporary Western settings. We have further developed the instrument and added a categorization into local services with or without gatekeeping and centralized services. These three categories allow exploration of the balance of resources between local and centralized services, making comparisons within and between countries possible. This ESMS-R-Local Service variable can then be used as a quality indicator for MHS systems in general, proposed to be used by MHS managers in evaluating the progress of their organizations. More research on the validity of the proposed classification is needed.

## Figures and Tables

**Table 1:** Distribution of service units (basic stable input of care) and mental health personnel (full time equivalent) to local or/and centralized services.

ESMS-R Main Branch	Local Services Without Gatekeeping	Local Services With Gatekeeping	Centralized Services	BSIC Found *n* (%)	Different MTC Found/Possible *
Information for care	7	2	0	22 (2)	6/9
Accessibility for care	3	2	0	6 (1)	1/5
Self-help and voluntary care	7	1	2	191 (19)	6/10
Outpatient care	3	19	2	279 (28)	17/24
Day care	4	6	12	157 (16)	17/22
Residential care	0	0	19	331 (34))	14/19
Different BSIC found *n* (%)	367 (37.2)	213 (21.6)	406 (41.2)	986	
Different MTC found / possible *	20/24	18/30	23/35		61/89
Percentage of personnel ** (%)	11	22	67		

* ESMS-R includes 89 categories of the main types of care (MTCs), of which our data includes 61. ** Personnel converted to full-time equivalents (FTE).

**Table 2:** The full time equivalent (FTE) personnel per 1000 adults (18+) by provider status (a.), organizational level (b.), local versus centralized services (c.).

Catchment Areas *	Länsi-Uusi-Maa	Lohja	Hyvin-Kää	Porvoo	Helsinki	Jorvi	Peijas	Kymen-Laakso	Eksote	Turku	Salo	Vakka-Suomi	Turun-Maa	Sum	Weighted Average	SD
Mental health index (Finland = 100)	92.3	94	92.9	89	90	77.2	89.6	106.2	102.7	109.7	101	102.9	101.3		96	8.9
Size of catchment areas adult (18+) population (2012)	35,296	70,379	139,734	74,611	501,929	230,005	187,332	143,265	109,379	151,616	128,039	81,392	18,200	1,871,178	128,000 (median)	122,759
Total personell (FTE)																
Total personnel FTE per 1000	3.64	4.10	2.8827	3.10	4.01	2.80	3.46	4.17	2.98	4.82	3.31	3.67	2.93	3.63		0.61
a) Providers status																
Public personnel FTE per 1000	2.58	2.43	1.9642	1.52	2.69	2.06	2.51	2.38	2.43	2.58	2.03	1.87	1.94		2.35	0.35
Third sector personnel FTE per 1000	0.19	0.43	0.3766	0.97	1.26	0.38	0.39	1.11	0.14	0.95	0.59	0.18	0.00		0.73	0.40
Private company personnel FTE per 1000	0.87	1.24	0.5419	0.61	0.05	0.36	0.56	0.68	0.41	1.29	0.69	1.62	0.99		0.55	0.43
b) Organizational level														6784.72	3.62	
Primary health care personnel FTE per 1000	0.59	1.31	1.3203	1.14	1.28	2.05	2.04	0.72	0.23	3.22	1.90	1.85	0.28			
Primary healthcare personnel FTE per 1000	2.14	2.24	1.2661	1.92	1.87	1.30	1.78	2.37	1.01	2.51	1.98	2.37	1.22		1.83	0.49
Secondary healthcare personnel FTE per 1000	1.50	1.87	1.6165	1.17	2.14	1.50	1.68	1.80	0.18	2.31	1.33	1.30	1.71		1.69	0.52
Integrate primary and secondary healthcare personnel FTE per 1000									1.79					195.61		
c) Local vs centralized service level																
Local without gatekeeping FTE per 1000	0.51	0.33	0.4835	0.20	0.22	0.17	0.37	0.06	0.25	0.92	1.01	0.71	0.77		0.46	0.30
Local with gatekeeping FTE per 1000	0.46	0.90	0.6343	0.44	1.07	0.80	0.97	1.05	0.78	0.78	0.41	0.29	0.00		0.66	0.32
Total local resources (without and with gatekeeping) per 1000	0.97	1.23	1.1178	0.64	1.29	0.97	1.33	1.11	1.03	1.70	1.41	1.00	0.77		1.12	
Centralized FTE per 1000	2.67	2.87	1.7648	2.45	2.72	1.83	2.12	3.06	1.95	3.12	1.89	2.67	2.17		2.40	0.48
Relation of local resources																
% of local without gatekeeping FTE from total local FTE (per 1000)	53%	27%	0.4325	0.32	0.17	0.18	0.28	0.05	0.24	0.54	0.71	0.71	1.00		0.41	
% of local FTE from total resources (per 1000)	27%	30%	0.3878	0.21	0.32	0.35	0.39	0.27	0.34	0.35	0.43	0.27	0.26		0.31	

* The numbers of catchment areas indicate the data collection order, used in previous articles [23,34].

**Table 3:** Correlations between allocated full time equivalents per 1000 18+ in local vs centralized services.

Spearman‘s Rho (*N* = 13 Catchment Area)		Total Local Personnel	Local Without Gatekeeping Personnel	Local with Gatekeeping Personnel	Centralized Personnel
Total personnel	Correlation Coefficient	0.063	0.058	0.408	0.911 **
	Sig. (2-tailed)	0.838	0.851	0.167	0.000
Total local personnel	Correlation Coefficient		0.960 **	−0.538	−0.056
	Sig. (2-tailed)		0.000	0.058	0.856
Local without gatekeeping personnel	Correlation Coefficient			−0.635 *	−0.044
	Sig. (2-tailed)			0.020	0.887
Local with gatekeeping personnel	Correlation Coefficient				0.300
	Sig. (2-tailed)				0.320

** Correlation is significant at the 0.01 level (2-tailed). * Correlation is significant at the 0.05 level (2-tailed).

**Table 4:** The proportion of local vs. centralized services by provider status (a) and vertical level of organization (b) (*N* = 985).

	Proportion of Local vs Centralized Services	Sum (BSIC)	BSIC %	Sum FTE/1000 18+	FTE %
a) Provider status (*N* = 985)					
Third sector (*n* = 450)	Local without gatekeeping	244	54.2%		
	Local with gatekeeping	70	15.6%		
	Centralized	136	30.2%		
	Sum	450	100%	0.73	20.1%
Public (*n* = 416)	Local without gatekeeping	122	29.3%		
	Local with gatekeeping	129	31.0%		
	Centralized	165	39.7%		
	Sum	416	100%	2.35	64.7%
Private (*n* = 119)	Local without gatekeeping	1	0.8%		
	Local with gatekeeping	14	11.8%		
	Centralized	104	87.4%		
	Sum	119	100%	0.55	15.2%
b) Organizational level (*N* = 985)					
	Local without gatekeeping	20	10.5%		
Primary care (*n* = 193)	Local with gatekeeping	76	39.8%		
	Centralized	95	49.7%		
	Sum	191	100%	1.83	50.4%
Secondary health care (*n* = 769)	Local without gatekeeping	337	43.8%		
	Local with gatekeeping	130	16.9%		
	Centralized	302	39.3%		
	Sum	769	100%	1.69	46.6%
Integrated health and social care (*n* = 25)	Local without gatekeeping	10	40.0%		
	Local with gatekeeping	7	28.0%		
	Centralized	8	32.0%		
	Sum	25	100%	0.10	2.8%
Total Sum by provider status	Local without gatekeeping	367	37.3%	0.46	11.0%
	Local with gatekeeping	213	21.6%	0.66	22.0%
	Centralized	405	41.1%	2.40	67.0%
Total Sum *N* = 985	Sum	985	100%	3.63	100%

BSIC = Basic Stable Imput of Car; i.e. the organizational units that provide the services. FTE: allocated/full time equivalents.

## Supplementary Materials

The following are available online at http://www.mdpi.com/1660-4601/15/6/1131/s1, Table S1, Specialists participating in to the Delphi-panel. Table S2, The new coding variable classification process on European Service Mapping Schedule-Revised (ESMS-R) mapping tree. Table S3, Recoded Different main type of cares by main main branches and found frequency of units (BSICs) after first consensus classification round (Quadrange classification). Table S4, The classification of found service units (BSIC) on local versus centralized categories per 1000 adults (18+). Table S5, Sosioeconomic indicators from catchment areas. Table S6, Linear regression modeling with total. Figure S1, ESMS-R (DESDE) mapping tree (Salvador-Carulla et al. 2013, Salvador-Carulla et al. 2015, Sa including new coding variable. Code 1 = local without gatekeeping MTC service, 2 = local with gatekeeping MTC service, 3 = centralized gatekeeping MTC service by referral or distance gatekeeping.
