# Supplementary material for: Identifying Local and Centralized Mental Health Services—The Development of a New Categorizing Variable
Source: Int J Environ Res Public Health. 2018 May 31;15(6):1131. doi: 10.3390/ijerph15061131 (PMC6025394; doi:10.3390/ijerph15061131)
Supplement: Supplementary file 1 [file ijerph-15-01131-s001.pdf]

Supplementary Table 2. The new coding variable classification process on European Service Mapping Schedule-Revised (ESMS-R) mapping tree.

| N= 89 Main type of care (MTC) | 1= Local, without gatekeeping | 2= Local with gatekeeping | 3= Centralized, without gatekeeping | 4= Centaralized, with gatekeeping | Individual classification (n/6) round 1. Agreement with proposal | Consensus (round 1.) | Consensus and final decisions after individual classification (round 2.), 4 MTC (on red) re-classification and Category 4 re-coding to 3 (Centralized ) |
|---|---|---|---|---|---|---|---|
| **Information (I) (N=9)** | | | | | | | |
| I1.1 | 6 | | | | 6/6 | 1 | 1 |
| I1.2 | | 6 | | | 6/6 | 2 | 2 |
| I1.3 | 6 | | | | 6/6 | 1 | 1 |
| I1.4 | | 6 | | | 6/6 | 2 | 2 |
| I1.5 | 6 | | | | 6/6 | 1 | 1 |
| I2.1 | 6 | | | | 6/6 | 1 | 1 |
| I2.1.1 | 6 | | | | 6/6 | 1 | 1 |
| I2.1.2 | 6 | | | | 6/6 | 1 | 1 |
| I2.2 | 6 | | | | 6/6 | 1 | 1 |
| **Accessibility to care (A) (N=5)** | | | | | | | |
| A1 | 6 | | | | 6/6 | 1 | 1 |
| A2 | | 6 | | | 6/6 | 2 | 2 |
| A3 | | 5 | | | 5/5 | 2 | 2 |
| A4 | 6 | | | | 6/6 | 1 | 1 |
| A5 | 6 | | | | 6/6 | 1 | 1 |
| **Sef-Help and voluntary care (S) (N=10)** | | | | | | | |
| S1.1 | 6 | | | | 6/6 | 1 | 1 |
| S1.2 | 6 | | | | 6/6 | 1 | 1 |
| S1.3 | 6 | | | | 6/6 | 1 | 1 |
| S1.4 | 6 | | | | 6/6 | 1 | 1 |
| S1.5 | | | | 6 | 6/6 | 4 | 3 |
| S2.1 | 6 | | | | 6/6 | 1 | 1 |
| S2.2 | | 6 | | | 6/6 | 2 | 2 |
| S2.3 | 6 | | | | 6/6 | 1 | 1 |
| S2.4 | 5 | | | | 5/5 | 1 | 1 |
| S2.5 | | | | 6 | 6/6 | 4 | 3 |
| **Outpatient care (O) (N= 24)** | | | | | | | |
| O1.1 | 2 | 3 | | 1 | | 2 | 2 |
| O1.2 | 2 | 2 | 2 | | | 1 | 1 |
| O2.1 | | 6 | | | | 2 | 2 |
| O2.2 | 1 | 4 | | 1 | | 2 | 2 |
| O3.1 | 1 | 1 | 4 | | | 3 | 3 (3/6) |
| O3.2 | 1 | 1 | 1 | 2 | | 3 | 3 (3/6) |
| O4.1 | | 6 | | | | 3 | 2 (6/6) |
| O4.2 | 1 | 1 | 4 | | | 3 | 2 (6/6) |
| O5.1.1 | | 5 | | 1 | | 2 | 2 |
| O5.1.2 | 1 | 4 | | 1 | | 2 | 2 |
| O5.1.3 | | 2 | | 3 | | 2 | 2 |
| O5.2.1 | | 5 | | 1 | | 2 | 2 |
| O5.2.2 | 1 | 4 | | 1 | | 2 | 2 |
| O5.2.3 | | 2 | | 4 | | 2 | 2 |
| O6.1 | | 6 | | | | 2 | 2 |
| O6.2 | 1 | 4 | | 1 | | 2 | 2 |
| O7.1 | 1 | 4 | | 1 | | 2 | 2 |
| O7.2 | 1 | 3 | | 2 | | 2 | 2 |
| O8.1 | 1 | 4 | | 2 | | 2 | 2 |
| O8.2 | | 2 | 1 | 2 | | 2 | 2 |
| O9.1 | 3 | 4 | | 1 | | 2 | 2 |
| O9.2 | 2 | 3 | | 2 | | 2 | 2 |
| O10.1 | 4 | 1 | | 2 | | 1 | 1 |
| O10.2 | 1 | 2 | 2 | 1 | | 1 | 1 |
| **Day care (D) (22)** | | | | | | | |
| D0.1 | | 2 | 1 | 3 | | 4 | 3 |
| D0.2 | | | 3 | 3 | | 4 | 3 |
| D1.1 | | 2 | 2 | 4 | | 4 | 3 |
| D1.2 | | 2 | | 4 | | 4 | 3 |
| D2.1 | | 2 | 1 | 2 | | 2 | 2 |
| D2.2 | | 2 | 1 | 3 | | 4 | 3 |
| D6.1 | | 4 | 1 | 1 | | 2 | 2 |
| D6.2 | | 3 | 2 | 1 | | 4 | 3 |
| D3.1 | | 3 | 1 | 2 | | 4 | 3 |
| D3.2 | 1 | 3 | 1 | 1 | | 4 | 3 |
| D7.1 | | 3 | 2 | 2 | | 4 | 3 |
| D7.2 | | 2 | 2 | 1 | | 4 | 3 |
| D4.1 | | 5 | | 1 | | 2 | 2 |
| D4.2 | | 1 | 2 | 3 | | 4 | 3 |
| D4.3 | 1 | 3 | 1 | 1 | | 2 | 2 |
| D4.4 | 3 | 2 | 1 | | | 1 | 1 |
| D8.1 | | 4 | | 2 | | 2 | 2 |
| D8.2 | | 1 | 1 | 4 | | 4 | 3 |
| D8.3 | 4 | | 1 | 1 | | 2 | 2 |
| D8.4 | 4 | | 1 | 1 | | 1 | 1 |
| D5 | | 5 | 1 | | | 1 | 1 |
| D9 | | 4 | 1 | 1 | | 1 | 1 |
| **Residential services R (N=19) MTC types on additional figure 1.** | | | | | 6/6 | 4 | 3 (N=19) |
| **All residental services were classified to the category 4, after round 2, to the category 3., MTC codes on additional figure 1.m residential services** | | | | | | | |

Statistical test not done related to the individual classifications guiding role and small numbers of classifications. There were 14 MTC:s under half (<3/6) agreement.

Table 1. Specialists participating in to the Delphi-panel

| *Work position (At the time)* | *Academic crentials* |
|---|---|
| Director of nursing (TA-N) | MNSc, PhD-student (chair of panel) |
| Research Professor (KW) | Associate professor, PhD, Specialist in Psychiatry |
| Psychiatrist (MS) | Specialist in Psychiatry |
| Medicine director (GJ) | Associate professor, PhD, Specialist in Psychiatry |
| Chief Medical Officer (SS) | Associate professor, PhD, Specialist in Psychiatry |
| Development chief (PN) | Master of psychologists, PhD-student |
| Project chief (RK) | PhD |
| Psychiatric nurse, Project coordinator (MV) | MNSc |
| Psychiatric nurse, Project coordinator (VM) | RN |
| Administrative head nurse, Project coordinator (OO) | PhD |
| Psychiatric nurse, Project coordinator (SG) | RN |

Supplementary Table 3. Recoded Different main type of cares by main main branches and found frequency of units (BSICs) after first consensus classification round (Quadrange classification)

| Main type of care (MTC N = 89) | **1 = local without gatekeeping** | **2 = local with gatekeeping** | **3 = centralized without gatekeeping** | **4 = centralized with gatekeeping** | **Total main type of care** |
|---|---|---|---|---|---|
| Information for care | **7** | **2** | | | 9 |
| Accessibility to care | **3** | **2** | | | 5 |
| Self-Help and voluntary care | **7** | **1** | | **2** | 10 |
| Outpatient care | **3** | **17** | **4** | | 24 |
| Day care | **4** | **6** | | **12** | 22 |
| Residental care | | | | **19** | 19 |
| Total (MTC N = 89) | **24** | **28** | **4** | **33** | 89 |
| Percent of recoded MTC | **27.0** | **31.5** | **4.5** | **37.1** | 100 |
| Frequency (BSIC N = 987) | **360** | **189** | **34** | **396** | 979 |
| Percent of found BSICs | **37 %** | **19 %** | **3 %** | **40 %** | 100 |

BSIC = Basic Stable Input of Care; i.e. the organizational units that provide the services. Missing 8 BSIC.
*Different MTC (main type of care) in ESMS-R (European Service Mapping Schedule-Revised) branches (N = 9)*

Supplementary Table 4. The classification of found service units (BSIC) on local versus centralized categories per 1000 adults (18+)

| Catchment Area | Population (18 +) | Local without gatekeeping BSICs | Local without gatekeeping BSICs per 1000 | Local with gatekeeping BSICs | Local with gatekeeping BSICs per 1000 | Centralized BSICs | Centalized BSICs per 1000 | Total number of BSICs | Total number of BSICs per 1000 |
|---|---|---|---|---|---|---|---|---|---|
| Länsi-Uusimaa (1) | 35 296 | 10.00 | 0.28 | 3.00 | 0.08 | 8.00 | 0.23 | 21.00 | 0.59 |
| Lohja (2) | 70 379 | 15.00 | 0.21 | 9.00 | 0.13 | 16.00 | 0.23 | 40.00 | 0.57 |
| Hyvinkää (3) | 139 734 | 37.00 | 0.26 | 11.00 | 0.08 | 41.00 | 0.29 | 89.00 | 0.64 |
| Porvoo (4) | 74 611 | 19.00 | 0.25 | 3.00 | 0.04 | 21.00 | 0.28 | 43.00 | 0.58 |
| Helsinki (5) | 501 928 | 49.00 | 0.10 | 57.00 | 0.11 | 90.00 | 0.18 | 196.00 | 0.39 |
| Jorvi (6) | 230 005 | 25.00 | 0.11 | 16.00 | 0.07 | 31.00 | 0.13 | 72.00 | 0.31 |
| Peijas (7) | 187 332 | 30.00 | 0.16 | 19.00 | 0.10 | 36.00 | 0.19 | 85.00 | 0.45 |
| Carea (8) | 143 210 | 28.00 | 0.20 | 37.00 | 0.26 | 50.00 | 0.35 | 115.00 | 0.80 |
| Eksote (9) | 107 612 | 28.00 | 0.26 | 14.00 | 0.13 | 26.00 | 0.24 | 68.00 | 0.63 |
| Turku (10) | 151 616 | 42.00 | 0.28 | 16.00 | 0.11 | 34.00 | 0.22 | 92.00 | 0.61 |
| Salon seutu (11) | 128 039 | 54.00 | 0.42 | 19.00 | 0.15 | 29.00 | 0.23 | 102.00 | 0.80 |
| Vakka-Suomi (12) | 81 391 | 21.00 | 0.26 | 9.00 | 0.11 | 18.00 | 0.22 | 48.00 | 0.59 |
| Turunmaa (13) | 18 199 | 9.00 | 0.49 | 0.00 | 0.00 | 4.00 | 0.22 | 13.00 | 0.71 |
| Total | 1 869 352 | 367.00 | 0.20 | 213.00 | 0.11 | 404.00 | 0.22 | 984.00 | 0.00 |
| Mean | 143 796 | 28.23 | 0.25 | 16.38 | 0.10 | 31.08 | 0.23 | 75.92 | 0.59 |

Supplementary Table 5. Sosioeconomic indicators from catchment areas

| Catchment area | Population (+18 year) | Mental health index (not age adjusted)* | Education index** | Un-employment % |
|---|---|---|---|---|
| Länsi-Uusimaa (1) | 35 296 | 92.3 | 3.0 | 7.2 |
| Lohja (2) | 70 379 | 94.0 | 3.2 | 7.1 |
| Hyvinkää (3) | 139 734 | 92.9 | 3.5 | 6.0 |
| Porvoo (4) | 74 611 | 89.0 | 3.3 | 7.1 |
| Helsinki (5) | 501 928 | 90.0 | 4.1 | 7.5 |
| Jorvi (6) | 230 005 | 77.2 | 4.6 | 5.5 |
| Peijas (7) | 187 332 | 89.6 | 3.4 | 8.0 |
| Carea (8) | 143 210 | 106.2 | 3.0 | 12.2 |
| Eksote (9) | 107 612 | 102.7 | 3.0 | 11.8 |
| Turku (10) | 151 616 | 109.7 | 3.7 | 12.9 |
| Salo (11) | 128 039 | 101.0 | 3.2 | 8.8 |
| Vakka-Suomi (12) | 81 391 | 102.9 | 3.2 | 7.1 |
| Turunmaa (13) | 18 199 | 101.3 | 3.2 | 6.0 |
| Mean | 143 796 (median 128 000) | 96.1 | 3.4 | 8.2 |
| SD | 122 760 | 8.9 | 0.5 | 2.5 |

Data Statistics Finland [R] THL (National Institute for Health and Welfare), SOTKAnet Statistics and Indicator Bank.

Areas 1-9 data from 2011 and Areas 10-13 from 2012.

* Mental health index (MHI) is calculated for each area using three years data on the suicides and suicides attemps, of persons eligble for special reimbursement for antipsychotic medication, and of persons on disability pension (18-64 years) due to mental disorders (https://www.sotkanet.fi)

** Education years after primary school e.g. high school, professional school and university

Supplementary Table 6. Linear regression modeling with total personnel per 1000 adults as the dependent variable

| Independent variables | Model 1 | Model 2 | Model 3 | Model 4 |
|---|---|---|---|---|
| % of total personnel in centralized services | 0.9 (2.9) | 1.1 (2.4) | 1.6 (3.1) | 2.3 (2.4) |
| Catchment area size | | 1.3 E-06 (.000) | | 1.9 E-06 (.000) |
| Mental Health Index | | | 0.18 (0.007)* | 0.02 (0.007)* |

* $p < 0.05$, ** $p < 0.01$

**Supplementary figure 1. ESMS- R (DESDE) mapping tree** (Salvador-Carulla et al. 2013, Salvador-Carulla et al. 2015) **including new coding variable.** Code 1 = local without gatekeeping MTC service, 2 = local with gatekeeping MTC service, 3 = centralized gatekeeping MTC service by referral or distance gatekeeping.

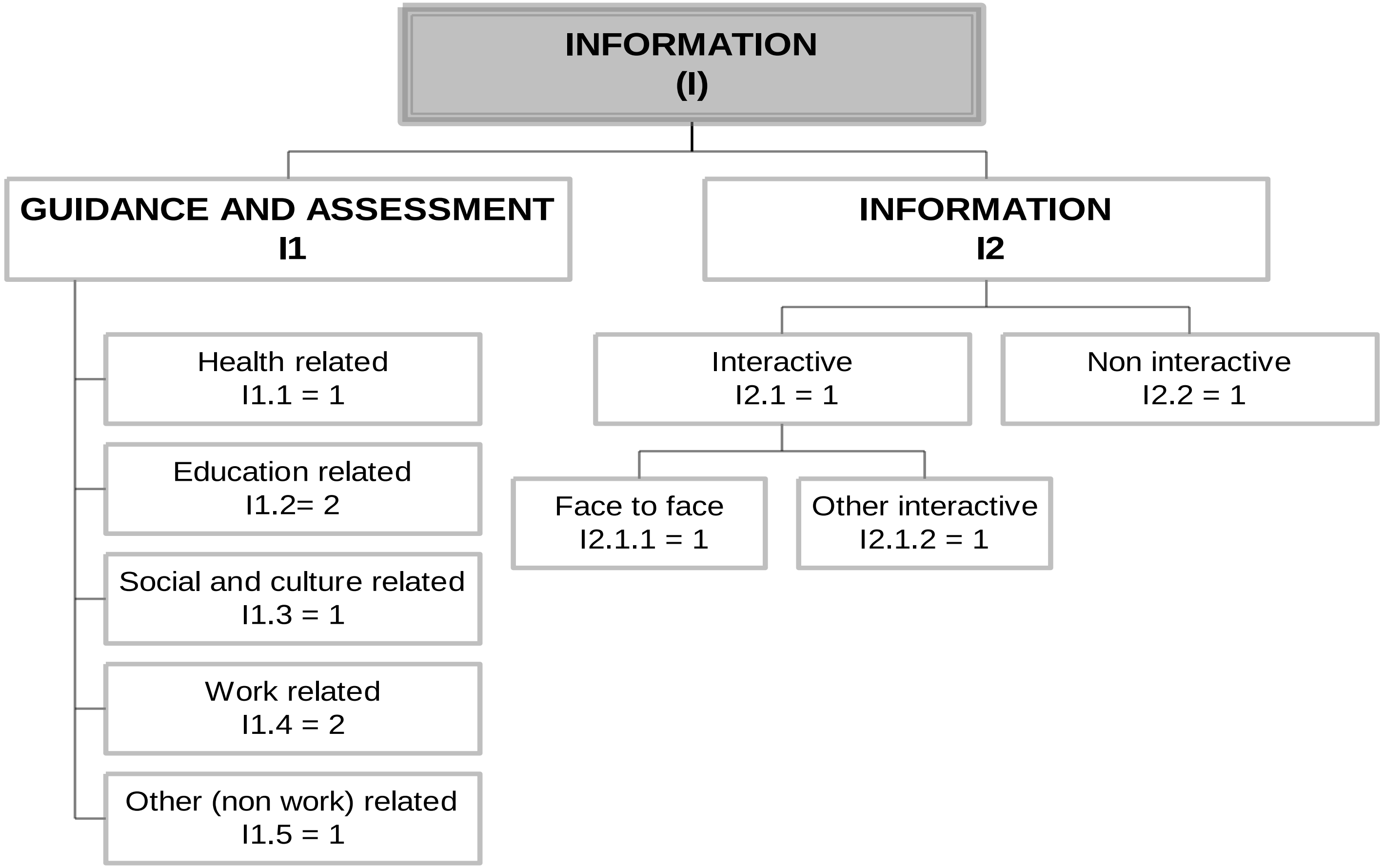

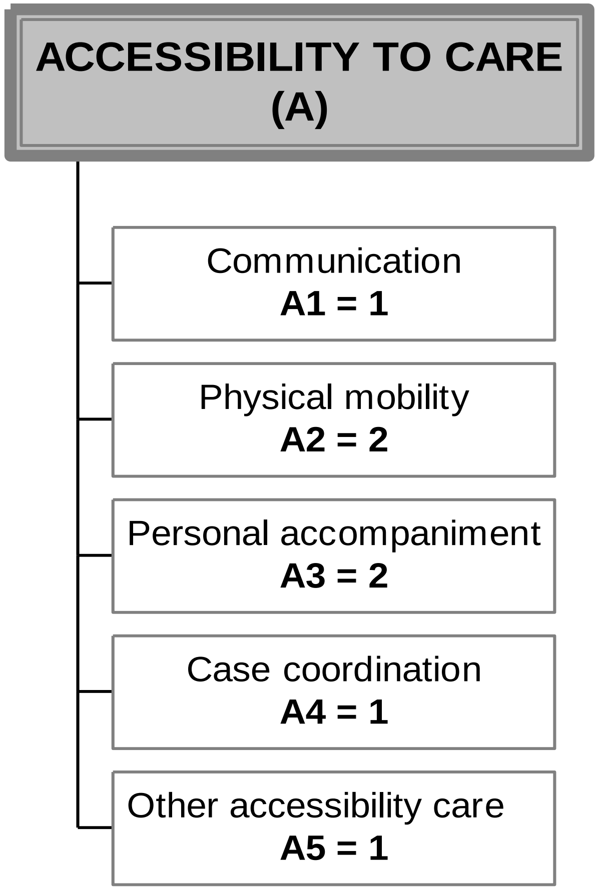

ACCESSIBILITY TO CARE (A)
Communication
A1 = 1
Physical mobility
A2 = 2
Personal accompaniment
A3 = 2
Case coordination
A4 = 1
Other accessibility care
A5 = 1

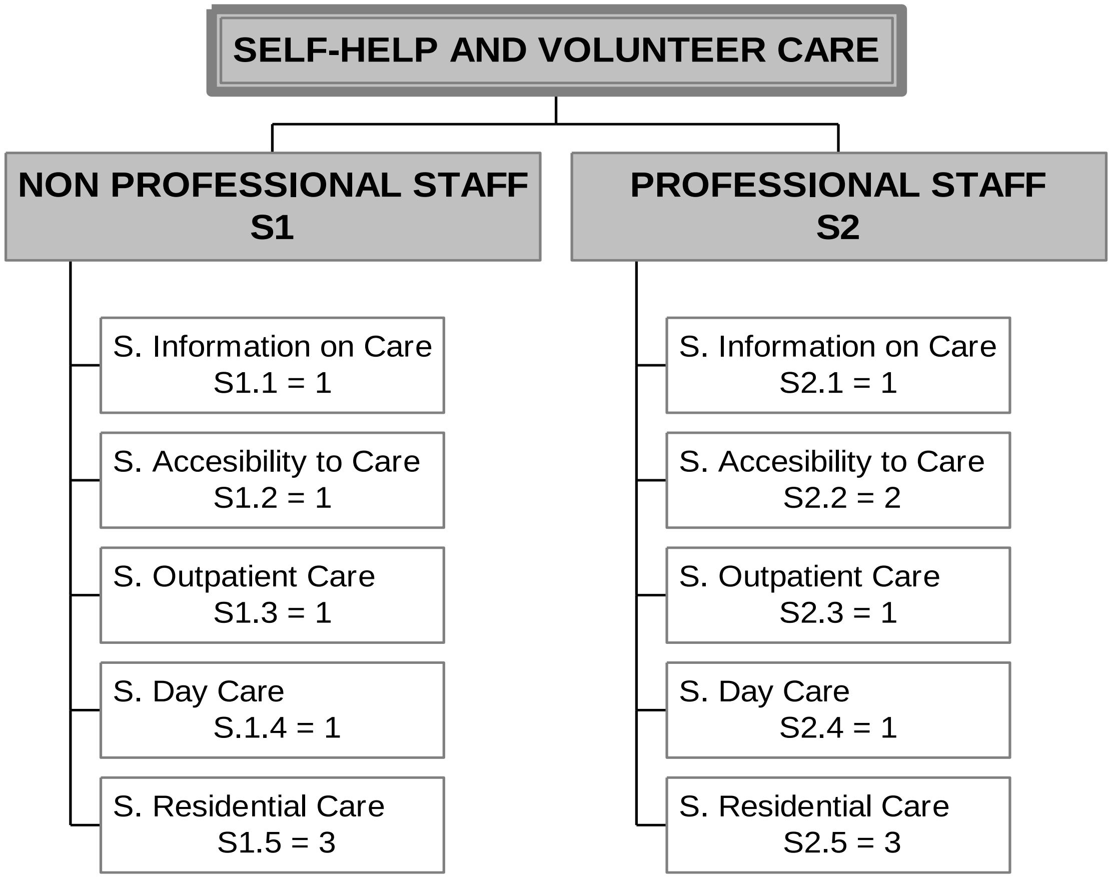
SELF-HELP AND VOLUNTEER CARE
NON PROFESSIONAL STAFF S1
PROFESSIONAL STAFF S2
S. Information on Care S1.1 = 1
S. Accesibility to Care S1.2 = 1
S. Outpatient Care S1.3 = 1
S. Day Care S.1.4 = 1
S. Residential Care S1.5 = 3
S. Information on Care S2.1 = 1
S. Accesibility to Care S2.2 = 2
S. Outpatient Care S2.3 = 1
S. Day Care S2.4 = 1
S. Residential Care S2.5 = 3

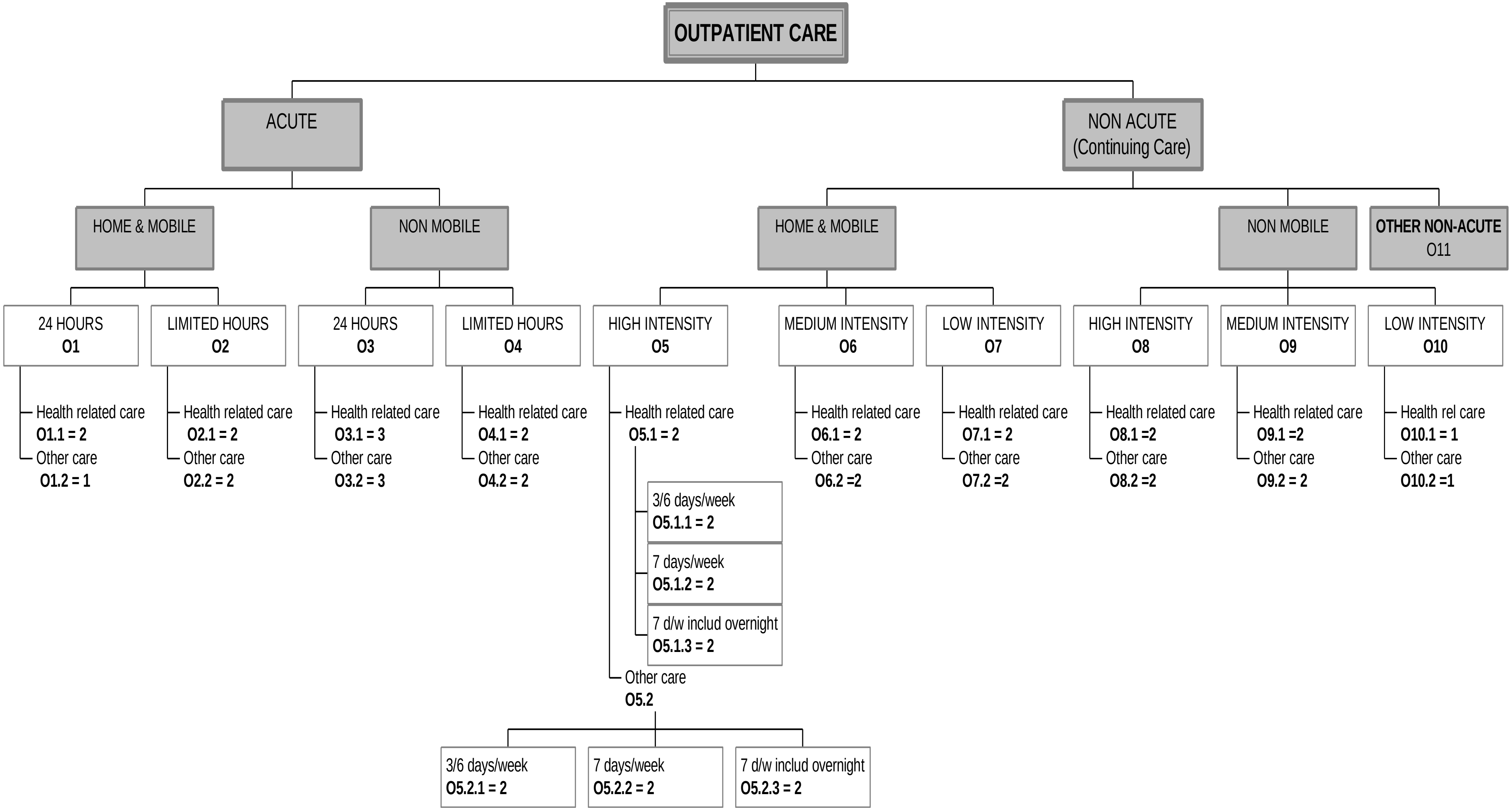

OUTPATIENT CARE
ACUTE
NON ACUTE
(Continuing Care)
HOME & MOBILE
NON MOBILE
HOME & MOBILE
NON MOBILE
OTHER NON-ACUTE
O11
24 HOURS
O1
LIMITED HOURS
O2
24 HOURS
O3
LIMITED HOURS
O4
HIGH INTENSITY
O5
MEDIUM INTENSITY
O6
LOW INTENSITY
O7
HIGH INTENSITY
O8
MEDIUM INTENSITY
O9
LOW INTENSITY
O10
Health related care
O1.1 = 2
Other care
O1.2 = 1
Health related care
O2.1 = 2
Other care
O2.2 = 2
Health related care
O3.1 = 3
Other care
O3.2 = 3
Health related care
O4.1 = 2
Other care
O4.2 = 2
Health related care
O5.1 = 2
3/6 days/week
O5.1.1 = 2
7 days/week
O5.1.2 = 2
7 d/w includ overnight
O5.1.3 = 2
Other care
O5.2
3/6 days/week
O5.2.1 = 2
7 days/week
O5.2.2 = 2
7 d/w includ overnight
O5.2.3 = 2
Health related care
O6.1 = 2
Other care
O6.2 =2
Health related care
O7.1 = 2
Other care
O7.2 =2
Health related care
O8.1 =2
Other care
O8.2 =2
Health related care
O9.1 =2
Other care
O9.2 = 2
Health rel care
O10.1 = 1
Other care
O10.2 =1

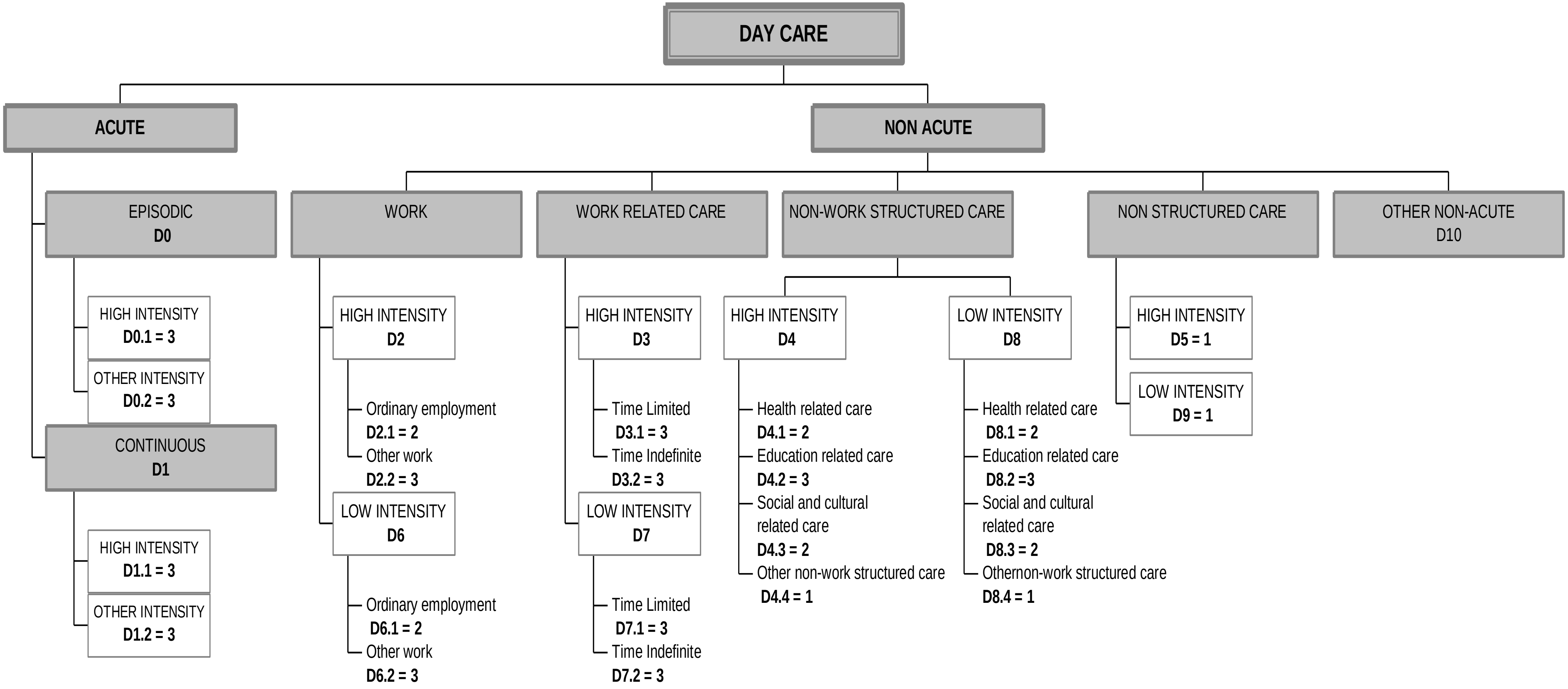
DAY CARE
ACUTE
NON ACUTE
EPISODIC
D0
HIGH INTENSITY
D0.1 = 3
OTHER INTENSITY
D0.2 = 3
CONTINUOUS
D1
HIGH INTENSITY
D1.1 = 3
OTHER INTENSITY
D1.2 = 3
WORK
HIGH INTENSITY
D2
Ordinary employment
D2.1 = 2
Other work
D2.2 = 3
LOW INTENSITY
D6
Ordinary employment
D6.1 = 2
Other work
D6.2 = 3
WORK RELATED CARE
HIGH INTENSITY
D3
Time Limited
D3.1 = 3
Time Indefinite
D3.2 = 3
LOW INTENSITY
D7
Time Limited
D7.1 = 3
Time Indefinite
D7.2 = 3
NON-WORK STRUCTURED CARE
HIGH INTENSITY
D4
Health related care
D4.1 = 2
Education related care
D4.2 = 3
Social and cultural related care
D4.3 = 2
Other non-work structured care
D4.4 = 1
LOW INTENSITY
D8
Health related care
D8.1 = 2
Education related care
D8.2 =3
Social and cultural related care
D8.3 = 2
Othernon-work structured care
D8.4 = 1
NON STRUCTURED CARE
HIGH INTENSITY
D5 = 1
LOW INTENSITY
D9 = 1
OTHER NON-ACUTE
D10

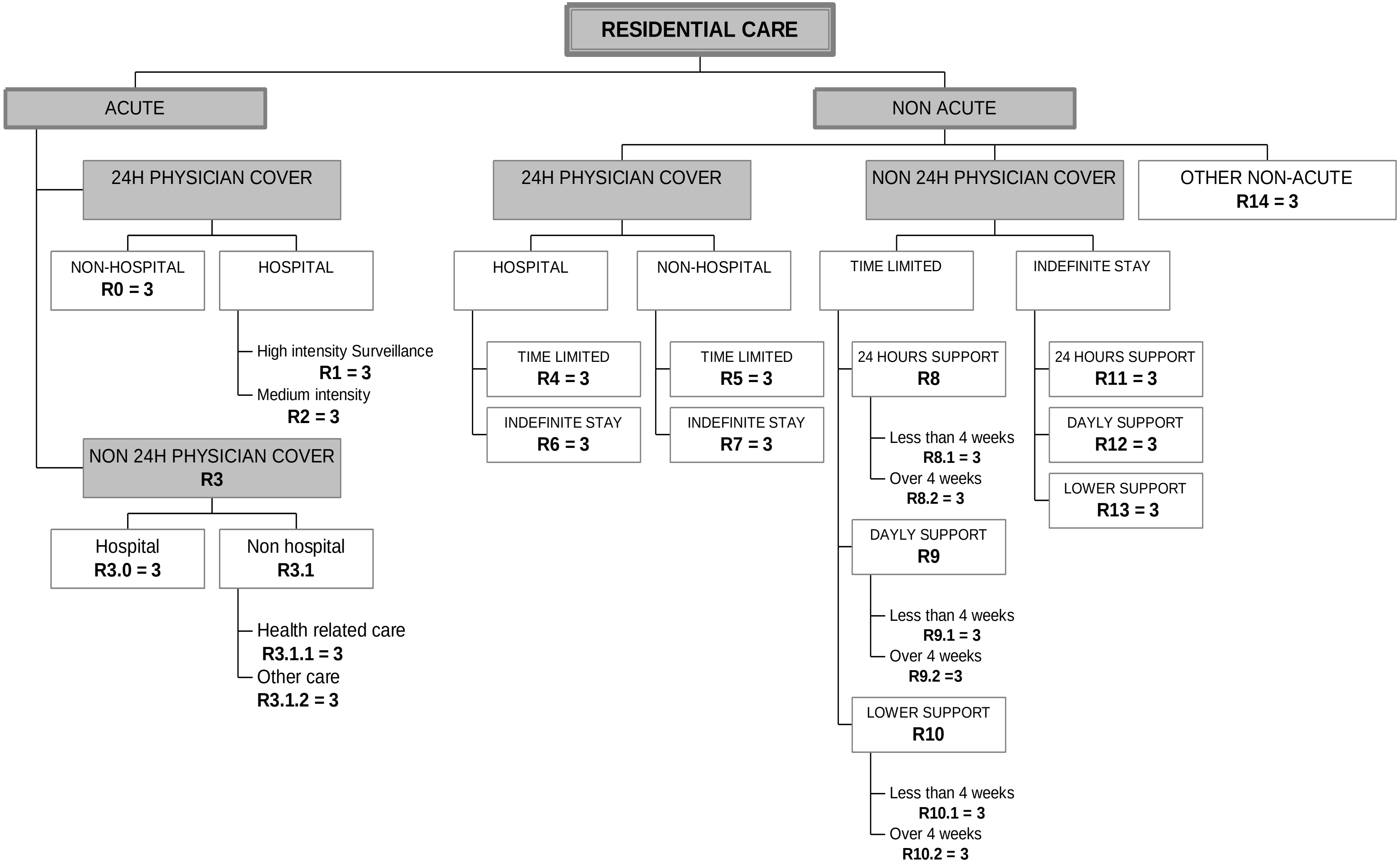
RESIDENTIAL CARE
ACUTE
24H PHYSICIAN COVER
NON-HOSPITAL
R0 = 3
HOSPITAL
High intensity Surveillance
R1 = 3
Medium intensity
R2 = 3
NON 24H PHYSICIAN COVER
R3
Hospital
R3.0 = 3
Non hospital
R3.1
Health related care
R3.1.1 = 3
Other care
R3.1.2 = 3
NON ACUTE
24H PHYSICIAN COVER
HOSPITAL
TIME LIMITED
R4 = 3
INDEFINITE STAY
R6 = 3
NON-HOSPITAL
TIME LIMITED
R5 = 3
INDEFINITE STAY
R7 = 3
NON 24H PHYSICIAN COVER
TIME LIMITED
24 HOURS SUPPORT
R8
Less than 4 weeks
R8.1 = 3
Over 4 weeks
R8.2 = 3
DAYLY SUPPORT
R9
Less than 4 weeks
R9.1 = 3
Over 4 weeks
R9.2 =3
LOWER SUPPORT
R10
Less than 4 weeks
R10.1 = 3
Over 4 weeks
R10.2 = 3
INDEFINITE STAY
24 HOURS SUPPORT
R11 = 3
DAYLY SUPPORT
R12 = 3
LOWER SUPPORT
R13 = 3
OTHER NON-ACUTE
R14 = 3
